# Brazil’s more doctors programme and infant health outcomes: a longitudinal analysis

**DOI:** 10.1186/s12960-021-00639-3

**Published:** 2021-08-14

**Authors:** Charlotte Bexson, Christopher Millett, Leonor Maria Pacheco Santos, Ricardo de Sousa Soares, Felipe Proenço de Oliveira, Thomas Hone

**Affiliations:** 1 School of Public Health, Imperial College London, London, UK; 2 Public Health Policy Evaluation Unit, Imperial College London, London, UK; 3 Department Public Health, University of Brasília, Brasília, DF Brazil; 4 Department of Health Promotion, Federal University of Paraíba, João Pessoa, PB Brazil

**Keywords:** Human resources for health, Brazil, Infant health, Maternal, newborn and child health, Primary care, Doctors

## Abstract

**Background:**

Providing sufficient numbers of human resources for health is essential for effective and accessible health services. Between 2013 and 2018, the Brazilian Ministry of Health implemented the *Programa Mais Médicos* (PMM) (More Doctors Programme) to increase the supply of primary care doctors in underserved areas of the country. This study investigated the association between PMM and infant health outcomes and assessed if heterogeneity in the impact of PMM varied by municipal socio-economic factors and health indicators.

**Methods:**

An ecological longitudinal (panel) study design was employed to analyse data from 5565 Brazilian municipalities over a 12-year period between 2007 and 2018. A differences-in-differences approach was implemented using longitudinal fixed effect regression models to compare infant health outcomes in municipalities receiving a PMM doctor with those that did not receive a PMM doctor. The impact of PMM was assessed on aggregate and in municipality subgroups.

**Results:**

On aggregate, the PMM was not significantly associated with changes in infant or neonatal mortality, but the PMM was associated with reductions in infant mortality rate (IMR) (of − 0.21; 95% CI: − 0.38, − 0.03) in municipalities with highest IMR prior to the programme’s implementation (where (IMR) > 25.2 infant deaths per 1000 live births). The PMM was also associated with an increase in the proportion of expectant mothers receiving seven or more prenatal care visits but only in municipalities with a lower IMR at baseline and high density of non-PMM doctors and community health workers before the PMM.

**Conclusions:**

The PMM was associated with reduced infant mortality in municipalities with the highest infant mortality rate prior to the programme. This suggests effectiveness of the PMM was limited only to the areas of greatest need. New programmes to improve the equitable provision of human resources for health should employ comprehensive targeting approaches balancing health needs and socio-economic factors to maximize effectiveness.

**Supplementary Information:**

The online version contains supplementary material available at 10.1186/s12960-021-00639-3.

## Background

An appropriate distribution of skilled human resources for health (HRH) is central to ensuring health systems meet population health needs and can improve health outcomes [1]. Effective and accessible health services, universal health coverage (UHC) [2] and the health-related Sustainable Development Goals (SDGs) all require trained and appropriately allocated HRH [3]. Globally, infant mortality remains a major challenge in many nations—over 25% of countries are not predicted to reach child-health SDG targets by 2030 [4]. By 2030 the WHO estimates the global needs-based shortage of healthcare workers will be above 14.5 million, of which nearly all will be required in low-and middle-income countries (LMICs) [3].

Brazil is an important country for studying HRH policies and infant health. The infant mortality rate (IMR) in 2019 was 12.4 per 1000 live births—above the average in upper middle-income and high-income countries (11.3 and 4.3 per 1000 live births respectively) [5]. However, this average conceals large geographical inequalities where northern states in Brazil such as Amapá and Maranhão have rates exceeding 20 infant deaths per 1000 live births. There have also been major health system strengthening efforts in Brazil including the *Estratégia de Saúde da Família* (ESF) (Family Health Strategy) which is a primary care service covering over 60% of the population [6]. The ESF includes multidisciplinary family health teams—consisting of a doctor, a nurse, a nurse assistant and community health workers (CHWs)—deployed to a defined local population [7]. Evidence demonstrates expansion of the ESF has been associated with reductions in adult [8] and infant mortality [9, 10], with one extra ESF doctor per 10,000 population associated with 7.08 fewer infant deaths per 10,000 live births [10]. However, ESF expansion has been constrained by the challenge of recruiting doctors to rural and underserved areas where there are poorer career prospects and working conditions [11].

To address HRH shortages in the ESF, the Brazilian government introduced the *Programa Mais Médicos* (PMM) (More Doctors Programme) in 2013. The initiative consisted of three strands: (i) an increase in the ‘emergency’ provision of doctors for primary care; (ii) reforms to increase the number of medical school places and primary care residency positions; and (iii) funds to improve and refurbish infrastructure in primary care facilities [11]. The emergency provision of doctors initially aimed to recruit Brazilian doctors to fill over 18,000 vacancies in underserved areas through higher salaries and training, but many posts remained unfilled [12]. Consequently, the Pan American Health Organisation (PAHO) facilitated an agreement between Cuba and Brazil to employ Cuban doctors in underserved areas. Within two years, the Brazilian Ministry of Health had positioned 17,625 predominantly Cuban doctors in underserved communities [11, 13]. It was initially planned PMM doctors would be allocated to municipalities based on federally-set criteria for prioritisation. These were: municipalities with 20% or more of the population living in extreme poverty; 100 municipalities with a population over 80,000 and the lowest income per capita; municipalities containing areas of extreme poverty; and municipalities with low human development index scores [14]. However, the prioritisation criteria was not adhered to well, and many non-criteria municipalities also received PMM doctors [14, 15].

Existing evaluations of the PMM have shown primary care coverage has increased [11], continuity and comprehensiveness of care have improved [16], hospitalisations for ambulatory care sensitive conditions (ACSC) have declined [13], and the programme has led to reductions in amenable adult mortality [14]. There is conflicting evidence on the PMM and child health outcomes as some studies have found the PMM was not associated with changes in IMR, infant birthweight or prenatal care appointments on average [17, 18]. However, one study found reductions in infant deaths in municipalities which did not have a doctor prior to the programme [19]. Another study found the PMM resulted in a shift in prenatal care provision from nurses to doctors without affecting infant health outcomes, and suggested nurses may be adequate substitutes for doctors in infant healthcare provision [18]. Despite this evidence, there has been little exploration of the heterogenous impacts of the PMM on maternal and infant health outcomes.

Given the large inequalities in healthcare services and population health across Brazil, understanding programme heterogeneity is crucial for informing policymakers and promoting efficient resource allocation [13]. This study assessed the association of the PMM on infant health outcomes, exploring whether the impact of the PMM varied by municipal socio-economic and health system characteristics. Models were adjusted for a range of socio-economic and health indicators that could confound the relationship between PMM and infant health outcomes. Additionally, the analytical strategy adjusts for time trends and time-invariant differences between municipalities. This allows the associations between PMM introduction and changes in infant health outcomes to be clearly identified.

## Methods

### Study design

A longitudinal (panel) study design was used to assess the association of PMM introduction and infant outcomes in Brazilian municipalities. The municipality was the unit of analysis with the dataset containing 5565 municipalities (out of a total of 5570 municipalities) with annual observations between 2007 and 2018. The application of doubly-robust inverse probability of treatment weighting with regression adjustment (IPTW-RA) adjusted for potential differences between PMM receiving and non-PMM receiving municipalities [20] (Additional file 1: Appendix S1).

### Data sources and variables

This study used collated data from the following websites of Brazilian government agencies and publicly available sources: the Brazilian Ministry of Health DATASUS website; the Instituto Brasileiro de Geografia e Estatística (IBGE) (Brazilian Institute for Geography and Statistics); the Ministério do Desenvolvimento Social e Combate à Fome) (MDS) (Ministry of Social Development and Fight Against Hunger); and the Sistema de Informações sobre Orçamentos Públicos em Saúde (SIOPS) (Information System for Public Health Budget) (Additional file 1: Appendix S2). All variables were obtained at the municipal level for each year between 2007 and 2018. Where data was not available annually (municipal socio-economic factors), linear interpolation was used for missing years.

The two primary outcome variables of interest consisted of municipal level IMR (deaths of infants under 1 year of age per 1000 live births), and neonatal mortality rate (NMR) (deaths of infants in first 28 days of life per 1000 live births).

An analytical framework (Additional file 1: Appendix S3) was developed to identify determinants of infant mortality and aid the selection of secondary outcomes and control variables. Seven intermediate outcomes were selected to reflect three pathways that influence infant health: (i) pre-delivery factors; (ii) delivery factors; and (iii) infant factors. Pre-delivery factors were captured with: the proportion of births with no prenatal care visits (%); the proportion of births with one to three prenatal care visits (%); the proportion of births with four to seven prenatal care visits (%); and the proportion of births with seven or more prenatal care visits (%). Hospital births were not analysed due to the high prevalence of hospital delivery in Brazil (in the 12-year period between 2007 and 2018, 97.6% of births in Brazil occurred in hospital). Infant factors were the proportion of newborns with very low birthweight (< 1500 g) (%); the proportion of newborns with low birth weight (< 2500 g) (%); and the number of infants hospitalised per 1000 live births.

The variable of interest was the density of PMM doctors per 1000 population in a municipality. This specification was chosen as it was superior to a binary treatment variable (denoting PMM uptake) as it allowed changes in PMM provision over time by municipality to be captured.

Regression models also included time-varying confounders to serve as proxies of socio-economic and health service that may be associated with infant health outcomes [13, 17, 18]. All variables measured in BRL were adjusted for inflation in the period between 2007 and 2018. These were: GDP per capita (Brazilian Real; BRL); income per capita (BRL); Gini index; proportion of households without adequate sanitation (%); proportion of households without electricity (%); proportion of population living in urban areas (%); proportion of the population with per capita income under 0.25 of the minimum wage (%); municipal illiteracy rate (aged 15 and below) (%); Bolsa Familia stipend per capita (BRL); municipal health spending per capita (BRL); private health insurance plan coverage (%); hospital bed density (beds per 1000 people); nurse density (nurses per 1000 people); mean mother’s age at birth; proportion of births to mothers with less than three years of education (%); proportion of births to mothers with four to seven years of education (%); proportion of births to mothers with eight to eleven years of education (%); and proportion of births to mothers with more than twelve years of education (%). Models also included municipal and year fixed effects.

### Statistical analysis

A differences-in-differences empirical strategy was used to explore the association between PMM implementation and infant health outcomes over time. The approach compares outcomes in participating municipalities (treatments) and non-participating municipalities (controls) before and after PMM implementation. IPTW-RA was used to balance treated and control municipalities on observed characteristics to reduce potential biases from unobserved selection bias [20]. This robust approach has been widely used to analyse the associated impact of the PMM on health outcomes [21].

Longitudinal regression models with a fixed effects specification were used to implement the differences-in-differences strategy. The fixed effect specification adjusts for municipal fixed effects (e.g. all time-invariant differences between municipalities) allowing only the associated changes within-municipalities over time to be estimated. Time (year) fixed effects were also included in the models to account for time trends and potential shocks. Fixed effects models are considered an effective way to address the hierarchical structure of panel data [13, 17, 18] and are an established tool for programme evaluations, including those assessing the impact of PMM [13, 17, 18].

Data analysis occurred in several stages. First, the data was presented descriptively including the mean, standard deviation and mean change for each variable between 2007 and 2018 presented by PMM receiving and non-PMM receiving municipalities. Secondly, the data was explored visually with maps showing the distribution of municipalities in which PMM doctors were introduced and graphs demonstrating parallel trends in IMR and NMR prior to PMM introduction. Thirdly, adjusted fixed effects regression models investigated the association between PMM introduction on each of the two primary outcome variables and the seven intermediate outcome variables. The association between the PMM and IMR and NMR for each of the years following PMM introduction was also investigated with dummy variables for each year post-PMM implementation. Separate regression models were undertaken for each variable separately adjusting for confounders, and time and year fixed effects. All models employed weighting from IPTW-RA.

Finally, heterogeneity in the association between PMM and outcomes was assessed by municipal socio-economic and healthcare characteristics. Municipalities were stratified by: urbanisation rates; poverty rates; IMR at baseline (2007); non-PMM public primary care doctor density prior to the PMM; nurse density prior to the PMM; CHW density prior to the PMM; and programme prioritisation. The association between the PMM and IMR and NMR for the year-by-year specification was also stratified by municipalities with the highest IMR at baseline.

## Results

**Fig. 1:**
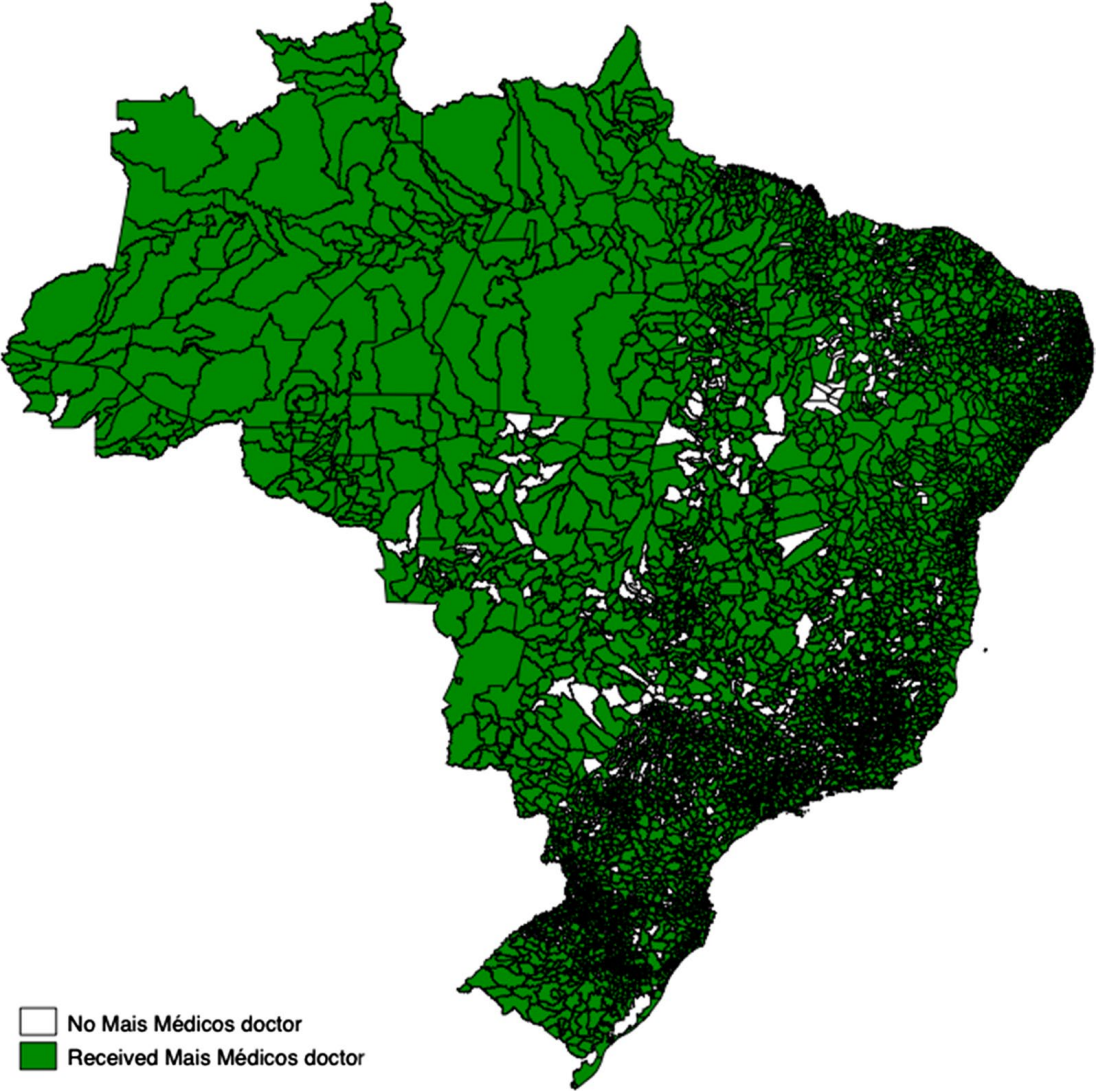
Allocation of PMM doctors in Brazilian municipalities, 2013–2018

**Fig. 2:**
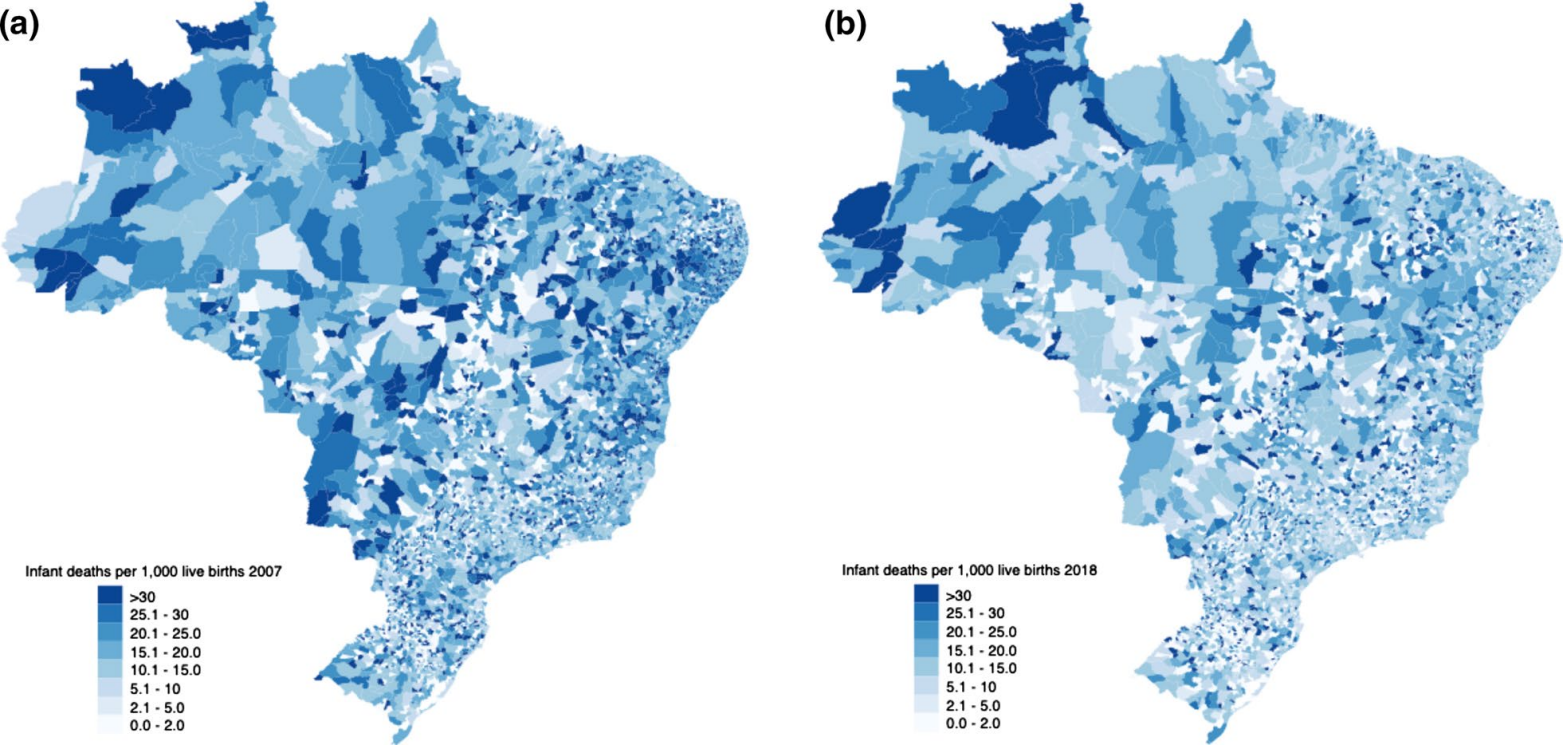
**a**, **b** Infant mortality rate in Brazilian municipalities, 2007 and 2018

Of the 5565 Brazilian municipalities examined, 4660 received PMM doctors between 2013 and 2018. (Fig. 1). Over the 12-year study period, there was a decrease in the mean municipal IMR from 16.48 in 2007 to 12.41 in 2018 (24.7% relative decrease), whilst the mean municipal NMR decreased from 11.13 to 8.78 over the same period (21.1% relative decrease) (Table 1; Fig. 2a and b). There was a general improvement in socio-economic characteristics across municipalities during the 12-year period, including an increase in GDP per capita (BRL 9172.81 in 2007 to BRL 23,165.27 in 2018), and an increase in municipal health spending per capita (BRL 270.02 in 2007 to BRL 378.62 in 2018). The means in socio-economic and health service provision characteristics of municipalities were similar for both PMM receiving and non-PMM receiving municipalities (Additional file 1: Appendix S4). Trends in mean rates of IMR and NMR in PMM receiving and non-PMM receiving municipalities between 2007 and 2018 illustrated parallel trends in the periods before PMM introduction (Additional file 1: Appendix S5, S6).

**Table 1:** Descriptive statistics for outcome variables and confounders

	Mean 2007	Mean 2018	Mean change (%)	Mean	SD
Population	33,072.34	37,456.95	13.26	35,526.03	209,427.40
GDP per capita (BRL)	9172.81	23,165.27	152.54	16,260.59	18,818.06
Income per capita (BRL)	447.13	617.66	38.14	532.38	268.23
Gini coefficient	0.51	0.45	− 11.76	0.48	0.08
Households with no electricity (%)	5.99	0.44	− 92.65	2.09	5.67
Households with inadequate sanitation (%)	10.45	7.58	− 27.46	8.65	13.32
Urbanisation rate (%)	62.76	66.31	5.66	64.60	22.16
Illiteracy rate (15 + years) (%)	17.82	11.73	− 34.18	14.77	9.54
Population with per capita income under 0.25 minimum wage (%)	28.49	11.20	− 60.69	19.28	17.88
Bolsa Familia stipend per capita (BRL)	72.17	219.89	204.68	156.34	132.05
Health expenditure per capita (BRL)	270.02	839.11	310.43	535.36	314.71
Private health insurance plans per capita	0.06	0.08	33.33	0.08	0.13
Hospital beds per 1000 population	1.93	1.55	− 19.69	1.71	2.08
Nurses per 1000 population	0.43	0.90	109.30	0.66	0.37
Births with low birthweight (%)	7.36	7.91	7.47	7.69	3.41
Births with very low birthweight (%)	0.94	1.10	17.02	1.03	1.21
Infants hospitalised per 1000 live births	206.18	197.45	− 4.23	190.64	118.63
Neonatal mortality rate (NMR)	11.13	8.78	− 21.11	9.77	11.27
Infant mortality rate (IMR)	16.48	12.41	− 24.70	14.09	13.68
Mean municipal mother’s age	24.80	26.35	6.25	25.46	1.49
Mothers with 0 to 3 years education (%)	13.80	3.05	− 77.90	7.60	7.76
Mothers with 4 to 7 years education (%)	36.38	18.02	− 50.47	27.34	11.34
Mothers with 8 to 11 years education (%)	35.47	62.06	74.96	50.12	14.69
Mothers with more than 12 years education (%)	11.14	15.31	37.43	12.61	7.97
No prenatal care (%)	1.43	1.32	− 7.69	1.71	3.21
1–3 prenatal care visits (%)	7.46	4.29	− 42.49	5.93	6.13
4–6 prenatal care visits (%)	34.74	20.14	− 42.03	27.20	15.08
7 or more prenatal care visits (%)	55.02	73.99	34.48	64.48	20.27

Sources: Brazilian Ministry of Health DATASUS website; Brazilian Institute for Geography and Statistics (IBGE); Ministry of Social Development and Fight Against Hunger (MDS); Information System for Public Health Budget (SIOPS); Mean refers to the average over the 12-year period between 2007 and 2018

In adjusted IPTW-RA panel regression models there was no significant association in changes in the density of PMM doctors and IMR or NMR (Table 2). Furthermore, there was no association between the PMM and IMR or NMR when the effect was tested for each year following PMM introduction (Additional file 1: Appendix S7). For intermediate outcomes there were no significant associations except for low birthweight births where a one unit increase in the number of doctors per 1000 population was associated with a very small 0.03 (95% CI: 0.00,0.05) percentage point increase in the proportion of infants with a low birthweight (Additional file 1: Appendix S8, S9).

**Table 2:** Effect of PMM density on infant mortality rate and neonatal mortality rate

	**IMR**	**95% CI**	**NMR**	**95% CI**
PMM density per 1000 population	− 0.01	− 0.11,0.09	− 0.01	− 0.09,0.08
GDP per capita (BRL)	− 0.00*	− 0.00,− 0.00	− 0.00	− 0.00,0.00
Income per capita (BRL)	0.00	− 0.00,0.01	0.00	− 0.00,0.01
Gini coefficient	− 3.09	− 10.12,3.94	− 2.57	− 8.33,3.19
Households with inadequate sanitation (%)	− 0.02	− 0.05,0.02	− 0− 0.02	− 0.05,0.01
Households with no electricity (%)	− 0.01	− 0.07,0.05	− 0.02	− 0.07,0.03
Urbanisation rate (%)	0.03	− 0.01,0.07	0.01	− 0.02,0.04
Illiteracy rate (15 + years) (%)	0.03	− 0.13,0.19	0.01	− 0.11,0.14
Population with per capita income under 0.25 minimum wage (%)	0.06*	0.01,0.11	0.4	− 0.00,0.09
Bolsa Familia stipend per capita (BRL)	0.00*	0.00,0.01	0.00	− 0.00,0.00
Health expenditure per capita (BRL)	0.00**	0.00,0.00	0.00	− 0.00,0.00
Private health insurance plans per capita	0.30	− 1.20,1.79	− 0.01	− 1.55,1.53
Hospital beds per 1000 population	0.12	− 0.05,0.29	0.08	− 0.05,0.22
Nurses per 1000 population	− 0.41	− 1.19,0.38	− 0.60	− 1.24,0.03
Mean municipal mother’s age	0.21	− 0.04,0.47	0.08	− 0.15,0.31
Births to women with 0–3 years of education	0.04	− 0.00,0.08	0.01	− 0− 0.02,0.05
Births to women with 4–7 years of education	0.02	− 0.01,0.04	0.02	− 0.01,0.05
Births to women with 8–11 years of education	− 0.01	− 0.03,0.02	− 0.00	− 0.02,0.02
Births to women with 12 or more years of education	− 0.03	− 0.07,0.01	− 0.02	− 0.06, 0.02
N (municipalities)	5565		5565	
N (observations)	66,778		66,778	

* *p* < 0.05, ** *p* < 0.01, *** *p* < 0.001. All models applied inverse probability of treatment weighting with regression adjustment (IPTW-RA). Primary outcome variables: infant mortality rate (IMR) and neonatal mortality rate (NMR). Intermediate outcome variables: infants hospitalised per 1000 live births; seven or more prenatal care visits; and proportion of infants born with a low birthweight (< 2500 g). Models adjusted for GDP per capita (BRL), income per capita (BRL), Gini coefficient, proportion of households with inadequate sanitation (%), proportion of households with no electricity (%), proportion of population living in urban areas (%), proportion of population illiterate above the age of 15 (%), proportion of the population with per capita income under 0.25 minimum wage (%), Bolsa Familia stipend (BRL), private health insurance plans per capita, heath expenditure per capita (BRL), hospital beds per 1000 population, nurses per 1000 population, mean municipal mother’s age, proportion of mothers with zero to three years of education (%), proportion of mothers with four to seven years of education (%), proportion of mothers with eight to eleven years of education (%), proportion of mothers with more than twelve years of education (%), and municipality and time fixed effects

Municipalities were stratified by their urbanisation rate to explore potential heterogeneity. The PMM was not associated with changes primary or intermediate outcomes in any grouping of municipalities by urbanisation (Additional file 1: Appendix S10). Similarly, there were predominantly non-significant associations from PMM expansion when municipalities were stratified by poverty rates (Additional file 1: Appendix S11). However, in low-poverty municipalities (less than 3.69% of the population lived in poverty) a one unit increase in PMM doctors per 1000 was associated with an increase of 1.64 infant hospitalisations (95% CI: 0.08,3.21) per 1000 live births, and a 0.08 percentage point decrease (95% CI: − 0.01, − 0.14)in the proportion of infants born with low birthweight.

Municipalities were stratified by baseline (2007) IMR into quintiles (Table 3). Most outcomes were non-significant across the strata, however a one unit increase in PMM doctors per 1000 was associated with a decrease in the IMR (of − 0.21; 95% CI: − 0.38, − 0.03) in municipalities with the highest IMR at baseline (Q5; IMR > 25.2). In municipalities with highest IMR at baseline, a one unit increase in PMM doctors per 1000 was associated with a decrease in the IMR immediately following PMM introduction (− 0.61; 95% CI: − 0.99, − 0.24) and one year after PMM introduction (− 0.31; 95%CI: − 0.58, − 0.05). However, significant associations were not found after two years post introduction (Additional file 1: Appendix S12). Municipalities with lower IMR at baseline saw increases in the proportion of births with mothers receiving seven or more prenatal care visits, specifically in municipalities with 0 IMR per 1000 live births (0.09; 95% CI: 0.01,0.17) and 1.9–12.2 deaths per 1000 live births (0.60; 95% CI: 0.13,1.08).

**Table 3:** Effect of PMM density on primary and intermediate outcomes for subgroups of municipal baseline IMR

	IMR	95% CI	NMR	95% CI	Infants hospitalised	95% CI	≤ 7 prenatal care visits	95% CI	Low birth weight	95% CI
Quintiles of baseline IMR (2007)										
Q1 (0 deaths)	− 0.01	− 0.14,0.13	− 0.01	− 0.12,0.09	0.38	− 0.57,1.32	0.09*	0.01,0.17	0.02	− 0.01,0.06
Q2 (1.89–12.2)	− 0.37	− 0.75,0.01	− 0.20	− 0.53,0.13	0.10	− 3.08,3.27	0.60*	0.13,1.08	0.07	− 0.01,0.16
Q3 (12.21–17.54)	0.07	− 0.27,0.42	0.12	− 0.15,0.39	1.36	− 0.51,3.24	0.34	− 0.11,0.79	0.08	− 0.02,0.17
Q4 (17.58–25.23)	0.10	− 0.21,0.41	0.12	− 0.14,0.38	− 0.10	− 2.53,2.32	0.05	− 0.21,0.31	− 0.01	− 0.08,0.05
Q5 (25.24–209.3)	− 0.21*	− 0.38,− 0.03	− 0.14	− 0.30,0.02	− 0.64	− 2.36,1.07	− 0.03	− 0.16,0.11	0.03	− 0.02,0.07

* *p* < 0.05, ** *p* < 0.01, *** *p* < 0.001. All models applied inverse probability of treatment weighting with regression adjustment (IPTW-RA). Primary outcome variables: infant mortality rate (IMR) and neonatal mortality rate (NMR). Intermediate outcome variables: infants hospitalised per 1000 live births; seven or more prenatal care visits; and proportion of infants born with a low birthweight (%). Models adjusted for GDP per capita (BRL), income per capita (BRL), Gini coefficient, proportion of households with inadequate sanitation (%), proportion of households with no electricity (%), proportion of population living in urban areas (%), proportion of population illiterate above the age of 15 (%), proportion of the population with per capita income under 0.25 minimum wage (%), Bolsa Familia stipend (BRL), private health insurance plans per capita, heath expenditure per capita (BRL), hospital beds per 1000 population, nurses per 1000 population, mean municipal mother’s age, proportion of mothers with zero to three years of education (%), proportion of mothers with four to seven years of education (%), proportion of mothers with eight to eleven years of education (%), proportion of mothers with more than twelve years of education (%), and municipality and time fixed effects

Municipalities were also stratified by HRH densities prior to the PMM. Generally, most outcomes and strata of HRH were non-significant suggesting little evidence of heterogenous associations between the PMM and infant health outcomes across municipality subgroups by HRH densities (Additional file 1: Appendix S13–S15). For municipalities with the highest densities of non-PMM doctors and community health workers prior to the programme, the PMM was associated with small increases in the proportion of births with mothers receiving seven or more prenatal care visits. Additionally, when heterogeneity in the impact of PMM was explored by programme prioritisation criteria, there were not significant associations for IMR and NMR.

## Discussion

This study found the PMM was not associated with a reduction in IMR or NMR on average, however there were small associated reductions in IMR in municipalities with high IMR prior the programme. There appeared to be little effect of the PMM on other outcomes or across socio-economic strata of municipalities, including programme prioritisation criteria.

The general absence of an impact of the PMM on aggregate corroborates previous studies on the PMM [17, 18], yet is in contrast to evidence from the ESF in Brazil [10] that shows primary care doctor density is inversely associated with infant mortality. There are multiple reasons that could explain the lack of association. Firstly, the health impacts of the PMM may take longer to be realized. The PMM was implemented in 2013, and only five years of post-implementation data are used in this analysis. Furthermore, the additional number of doctors provided may have been too small to substantially impact infant health outcomes. Secondly, there is evidence that the PMM doctors were allocated to non-priority municipalities likely limiting their effectiveness and potential to reduce IMR [14, 15]. Many PMM doctors were allocated to areas with already low levels of IMR or where there were higher levels of non-PMM primary care doctors working. Diminishing returns from increasing doctor density may have also reduced the effectiveness of PMM doctors in decreasing IMR [10]. Thirdly, PMM doctors may have been limited in their ability to improve infant health given the large roles other health professionals (such as nurses and community health workers) play in the Brazilian health system and also the reliance on hospitals for birth.

This study found a lack of association between the PMM and infant health outcomes across strata when stratifying by municipal socio-economic characteristics including poverty and urbanisation. This suggests despite poor targeting of PMM doctors to priority municipalities [14, 15], the relationship between the PMM and IMR was not substantially affected by municipal socio-economic factors other than infant mortality. This is generally supported by other studies which report no association between PMM and infant mortality in municipality subgroups of mother’s education, mother’s age and marital status [18]. However, it does appear that baseline IMR is a determinant of the relationship between the PMM and IMR as there were IMR reductions associated with the PMM in municipalities with the highest IMR at baseline. This finding corroborates the results of research on the ESF [9] which found IMR reductions from ESF expansion were greatest in municipalities with the highest IMR. This finding suggests that PMM doctors may deliver health benefits where underlying health outcomes are poor and also programmes such as the PMM can contribute to improvements in health inequalities when targeting is based on health indicators rather than socio-economic factors.

The PMM was not associated with changes in prenatal care outcomes in most models. The PMM was associated with an increase in the proportion of expectant mothers receiving over seven prenatal care visits but only in municipalities with the lowest IMR at baseline and the highest density of CHWs and non-PMM doctors in 2012. This finding indicates wealthier municipalities with a higher density of HRH prior to the programme may have more effectively integrated PMM doctors to local health services, allowing nurses and CHWs to focus on prenatal care provision. Therefore, the PMM can introduce minor improvements to services and processes, but in areas that are considered least in need of new doctors.

This study has several limitations. First, the data collated from the Brazilian Ministry of Health and publicly available sources may have administrative errors—including possible underreporting of infant deaths in some municipalities. However, the data was collated from established sources which have been used for several evaluations of the PMM programme [13, 17, 18]. Additionally, statistical approaches employing time and municipal fixed effects would have likely accounted for some sources of bias. Second, the absence of adequate reporting systems for maternal and health outcomes in some municipalities prior to PMM may have skewed findings. Evidence demonstrates the expansion of health services in Brazil has reduced under-reporting and therefore expansion of health professionals could be associated with increases in mortality rates [22]. This may have masked some of the associations between the PMM and reductions in IMR. Third, although the data includes the entire five-year period of PMM implementation (2013–2018), the programme may have long-term impacts on infant health beyond 2018 which cannot be measured with this data. Fourth, the ecological design of the study restricts causal inference and prevents the exploration of heterogeneity within municipalities. Fifthly, there may have been systematic differences between PMM- recipient and non-recipient municipalities that could have biased the findings, although the use of IPTW-RA aimed to minimize these biases and represent the most robust method for observational studies.

The impact of the PMM in municipalities with high IMR at baseline indicates that HRH programmes can deliver improvements where the health needs are the greatest. There are wider policy implications from this work. More comprehensive targeting arrangements are necessary to maximize the health gains of scarce resources—both in Brazil and in other settings. This includes up-to-date data on health outcomes and socio-economic characteristics of local populations. Evidence indicates the effectiveness of the PMM was diluted due to inappropriate targeting of PMM doctors to the most needed areas [14]. The results from this study also suggest that the targeting criteria that were in place were of limited benefit as no effect of the PMM was found when stratifying by criteria. Policymakers may need to balance different and conflicting health gains from different sub-populations (e.g. infant health gains versus morbidity improvements in older populations) when distributing resources. Additionally, PMM doctors were often allocated to the most deprived regions within municipalities [14], implying that municipal-level health metrics and impact studies (including this one) are of only limited value for effective resource targeting. More detailed analyses on the sub-populations within municipalities most benefitting from the PMM is necessary to understand the complexity of the impact of PMM on a localised level.

In August 2019, President Jair Bolsonaro announced the creation of the *Médicos pelo Brasil* (Doctors for Brazil) Program, to replace the PMM. However as of March 2021, the new program has not yet been implemented and longer-term strategies to address the lack of primary care doctors in underserved areas are absent. Furthermore, the increased pressure on Brazil’s healthcare system due to COVID-19 led the federal government to publish new calls for PMM doctors in 2020, including in non-priority municipalities. Under the original PMM, there was a planned expansion of 15,000 extra medical schools places to train primary care doctors, who, in 2021 would be filling unserved positions in primary care. However, these extra places were not fully expanded and coupled with the expansion of PMM positions into non-priority municipalities, the challenges in the provision and distribution of primary care doctors in Brazil remain. Tackling the ongoing lack of doctors and their inappropriate distribution is key for further strengthening the health system, providing access to high-quality care, and tackling the large social inequalities that exist in the country.

## Conclusion

The PMM was associated with limited improvements in infant health, with only some small reductions in infant deaths in the areas with the highest infant mortality rates. Health gains can be achieved with the expansion of health professionals in the most vulnerable areas indicating comprehensive and robust targeting approaches are necessary for maximizing health gains.

## Supplementary Information

**Additional file 1.** Additional tables.

## Data Availability

All data used in this study are available from public sources (see citations in text). Data used that support the findings of the current study available from the corresponding author on reasonable request.

## Abbreviations

ACSC: Ambulatory care sensitive conditions
BRL: Brazilian Real
CHW: Community health worker
CI: Confidence Interval
ESF: Estratégia Saúde Família (Family Health Strategy)
GDP: Gross domestic product
HRH: Human resources for health
IBGE: Instituto Brasileiro de Geografia e Estastíca (Brazilian Institute for Geography and Statistics)
IMR: Infant mortality rate
IPTW-RA: Inverse probability of treatment weighting with regression adjustment
LMIC: Low-and middle-income country
MDS: Ministério do Desenvolvimento Social e Combate à Fome (Ministry of Social Development and Fight Against Hunger
MPB: Médicos Pelo Brasil (Doctors for Brazil)
NMR: Neonatal mortality rate
PAHO: Pan American Health Organisation
PMM: Programa Mais Médicos (More Doctors Programme)
SD: Standard Deviation
SDG: Sustainable Development Goal
SIOPS: Sistema Informações sobre Orçamentos Públicos em Saúde (Information System for Public Health Budget)
UHC: Universal health coverage
WHO: World Health Organisation

## References

[1] Campbell J, Buchan J, Cometto G, David B, Dussault G, Fogstad H, Fronteira I, Lozano R, Nyonator F, Pablos-Méndez A (2013). Human resources for health and universal health coverage: fostering equity and effective coverage. Bull World Health Organ.

[2] Pan American Health Organization. Universal Health Coverage. https://www.paho.org/hq/index.php?option=com_content&view=category&layout=blog&id=6253&Itemid=40690&lang=en. Accessed 08 Mar 2021.

[3] World Health Organization. Health workforce requirements for universal health coverage and the sustainable development goals: Background paper No.1 to the Global Strategy of Human Resources for Health. Geneva: World Health Organisation (WHO); 2016.

[4] Lozano R, Fullman N, Abate D, Abay SM, Abbafati C, Abbasi N (2018). Measuring progress from 1990 to 2017 and projecting attainment to 2030 of the health-related Sustainable Development Goals for 195 countries and territories: a systematic analysis for the Global Burden of Disease Study 2017. Lancet.

[5] World Bank. Mortality rate, infant (per 1,000 live births), High income, Upper middle income. 2019. https://data.worldbank.org/indicator/SP.DYN.IMRT.IN?locations=XD-XT. Accessed 10 Jan 2021.

[6] Macinko J, de Souza Mde F, Guanais FC, da Simoes CC (2007). Going to scale with community-based primary care: an analysis of the family health program and infant mortality in Brazil, 1999–2004. Soc Sci Med..

[7] Macinko J, Harris MJ (2015). Brazil's family health strategy — delivering community-based primary care in a universal health system. N Engl J Med.

[8] Hone T, Rasella D, Barreto ML, Majeed A, Millett C (2017). Association between expansion of primary healthcare and racial inequalities in mortality amenable to primary care in Brazil: a national longitudinal analysis. PLoS Med..

[9] Aquino R, de Oliveira NF, Barreto ML (2009). Impact of the Family Health Program on infant mortality in Brazilian municipalities. Am J Public Health.

[10] Russo LX, Scott A, Sivey P, Dias J (2019). Primary care physicians and infant mortality: Evidence from Brazil. PLOS ONE..

[11] Santos LMP, Oliveira A, Trindade JS, Barreto IC, Palmeira PA, Comes Y, Santos FO, Santos W, Oliveira JPA, Pessoa VM (2017). Implementation research: towards universal health coverage with more doctors in Brazil. Bull World Health Organ.

[12] Pereira L, Santos L, Santos W, Oliveira A, Rattner D (2016). Mais Médicos program: provision of medical doctors in rural, remote and socially vulnerable areas of Brazil, 2013–2014. Rural Remote Health.

[13] Maffioli EM, Hernandes Rocha TA, Vivas G, Rosales C, Staton C, Nickenig Vissoci JR (2019). Addressing inequalities in medical workforce distribution: evidence from a quasi-experimental study in Brazil. BMJ Glob Health..

[14] Hone T, Powell-Jackson T, Santos LMP, de Sousa SR, de Oliveira FP, Sanchez MN (2020). Impact of the Programa Mais médicos (more doctors Programme) on primary care doctor supply and amenable mortality: quasi-experimental study of 5565 Brazilian municipalities. BMC Health Serv Res.

[15] Özçelik EA, Massuda A, McConnell M, Castro MC (2021). Assessing the performance of beneficiary targeting in Brazil’s More Doctors Programme. Health Policy Plan.

[16] Comes Y, Trindade S, Pessoa VM, Barreto IC, Shimizu HE, Dewes D (2016). The implementation of the mais médicos (More doctors) program and comprehensiveness of care in the family health strategy. Ciencia e Saude Coletiva..

[17] Mattos E, Mazetto D (2019). Assessing the impact of more doctors’ program on healthcare indicators in Brazil. World Dev..

[18] Carrillo B, Feres J (2019). Provider supply, utilization, and infant health: Evidence from a physician distribution policy. Am Econ J Econ Pol.

[19] dos Santos J, dos Santos H, Dias C, Filho A (2020). Assessing the impact of a doctor in remote areas of Brazil. Int J Public Health..

[20] Austin PC, Stuart EA (2015). Moving towards best practice when using inverse probability of treatment weighting (IPTW) using the propensity score to estimate causal treatment effects in observational studies. Stat Med.

[21] Hone T, Saraceni V, Coeli CM, Trajman A, Rasella D, Millett C (2020). Primary healthcare expansion and mortality in Brazil’s urban poor: A cohort analysis of 1.2 million adults. PLoS Med..

[22] Rasella D, Aquino R, Barreto ML (2010). Impact of the Family Health Program on the quality of vital information and reduction of child unattended deaths in Brazil: an ecological longitudinal study. BMC Public Health.

