# Supplementary material for: Brazil’s more doctors programme and infant health outcomes: a longitudinal analysis
Source: Hum Resour Health. 2021 Aug 14;19:97. doi: 10.1186/s12960-021-00639-3 (PMC8363855; doi:10.1186/s12960-021-00639-3)
Supplement: Supplementary file 1 — Additional file 1. Additional tables. [file 12960_2021_639_MOESM1_ESM.docx]

**Additional material for Brazil’s More Doctors Programme and Infant Health Outcomes: A Longitudinal Analysis**

**Appendix S1. Sensitivity analysis using inverse probability of treatment weighting with regression adjustment (IPTW-RA).**

Weights for IPTW were generated using a logistic regression model on municipality treatment status (PMM receiving and non-PMM receiving) for adjusting for municipal-level variables: GDP per capita; mean income per capita; Gini index; municipal health spending per capita (BRL); municipal illiteracy rate (aged 15 and below) (%); Bolsa Familia stipend per capita (BRL); proportion of households without electricity (%); private health insurance plan coverage (%); proportion of population living in urban areas (%); proportion of the population with per capita income under 0.25 of the minimum wage (%); fertility rate; average years of schooling; unemployment rate of those aged 18 or older; population density (inhabitants per km^2^); population aged over 65 years (%); political affiliation concordance of mayor with federal government (yes/no); municipality in semiarid region (yes/no); municipality in amazon region (yes/no); primary care coverage (%); nurse density (nurses per 1,000); private doctors per 1,000; non-primary care public doctors per 1,000; hospital bed beds per 1,000; proportion of births to mothers with less than three years of education (%); proportion of births with 7 or more antenatal care consultations (%); preterm births (%); low birthweight rate (%); and infant mortality rate (2012).

A sensitivity analysis explored the effect of the PMM on infant mortality rate (IMR) with and without inverse probability of treatment weighting with regression adjustment (IPTW-RA).

Notes: * p<0.05, ** p<0.01, *** p<0.001; Sensitivity analysis investigates effect of PMM on infant mortality rate (IMR) with and without inverse probability of treatment weighting with regression adjustment (IPTW-RA). Models adjusted for GDP per capita (BRL), income per capita (BRL), Gini coefficient, proportion of households with inadequate sanitation (%), proportion of households with no electricity (%), proportion of population living in urban areas (%), proportion of population illiterate above the age of 15 (%), proportion of the population with per capita income under 0.25 minimum wage (%), Bolsa Familia stipend (BRL), private health insurance plans per capita, heath expenditure per capita (BRL), hospital beds per 1,000 population, nurses per 1,000 population, mean municipal mother’s age, proportion of mothers with zero to three years of education (%), proportion of mothers with four to seven years of education (%), proportion of mothers with eight to eleven years of education (%), proportion of mothers with more than twelve years of education (%), and municipality and time fixed effects.

|  | **IMR with  IPTW-RA** | **95% CI** | **IMR without IPTW-RA** | **95% CI** |
| --- | --- | --- | --- | --- |
| PMM density per 1,000 population | -0.01 | -0.11,0.09 | -0.01 | -0.06,0.04 |
| GDP per capita (BRL) | -0.00* | -0.00,-0.00 | -0.00* | -0.00,-0.00 |
| Income per capita (BRL) | 0.00 | -0.00,0.01 | 0.00* | 0.00,0.01 |
| Gini coefficient | -3.09 | -10.12,3.94 | -3.03 | -8.10,2.04 |
| Households with inadequate sanitation (%) | -0.02 | -0.05,0.02 | -0.02 | -0.05,0.02 |
| Households with no electricity (%) | -0.01 | -0.07,0.05 | -0.01 | -0.06,0.03 |
| Urbanisation rate (%) | 0.03 | -0.01,0.07 | 0.02 | -0.01,0.05 |
| Illiteracy rate (15+ years) (%) | 0.03 | -0.13,0.19 | 0.03 | -0.08,0.14 |
| Population with per capita income under 0.25 minimum wage (%) | 0.06* | 0.01,0.11 | 0.06** | 0.02,0.10 |
| Bolsa Familia stipend per capita (BRL) | 0.00* | 0.00,0.01 | 0.00** | 0.00,0.01 |
| Health expenditure per capita (BRL) | 0.00** | 0.00,0.00 | 0.00*** | 0.00,0.00 |
| Private health insurance plans per capita | 0.30 | -1.20,1.79 | 0.56 | -1.50,2.63 |
| Hospital beds per 1,000 population | 0.12 | -0.05,0.29 | 0.11 | -0.04,0.27 |
| Nurses per 1,000 population | -0.41 | -1.19,0.38 | -0.33 | -0.85,0.19 |
| Mean municipal mother’s age | 0.21 | -0.04,0.47 | 0.21** | 0.06,0.35 |
| Births to women with 0-3 years of education | 0.04 | -0.00,0.08 | 0.04* | 0.01,0.07 |
| Births to women with 4-7 years of education | 0.02 | -0.01,0.04 | 0.01 | -0.01,0.03 |
| Births to women with 8-11 years of education | -0.01 | -0.03,0.02 | -0.01 | -0.03,0.01 |
| Births to women with 12 or more years of education | -0.03 | -0.07,0.01 | -0.03* | -0.06,-0.00 |
|  |  |  |  |  |
| N (municipalities) | 5,565 |  | 5,565 |  |
| N (observations) | 66,778 |  | 66,778 |  |

**Appendix S2. Variable name and calculation by source**

| **Variable Name** | **Variable Calculation** | **Source** | **Link** |
| --- | --- | --- | --- |
| Population | Municipal population | IBGE – Municipal population estimates | [https://sidra.ibge.gov.br/ pesquisa/estimapop/tabelas](https://sidra.ibge.gov.br/pesquisa/estimapop/tabelas) |
| GDP | GDP per capita (BRL) | IBGE – Municipal GDP estimates | [https://sidra.ibge.gov.br/ pesquisa/pib-munic/tabelas](https://sidra.ibge.gov.br/pesquisa/pib-munic/tabelas) |
| Income | Income per capita (BRL) | IBGE – Municipal demographic estimates* | [https://sidra.ibge.gov.br/pesquisa /censo-demografico/demografico-2010/inicial](https://sidra.ibge.gov.br/pesquisa/censo-demografico/demografico-2010/inicial) |
| Gini | Municipal Gini index |  |  |
| Poor | Percentage of population with per capita income under 0.25 minimum wage (%) |  |  |
| Illiterate | Percentage of population who are illiterate aged over 15 years (%) |  |  |
| Urban | Percentage of the population living in urban area (%) |  |  |
| Sanitation | Percentage of population without adequate sanitation (%) |  |  |
| Electricity | Percentage of population with no electricity (%) |  |  |
| Live births | Live births | Ministry of Health DATASUS - SIM | [http://www2.datasus.gov.br /DATASUS/index.php?area=0205](http://www2.datasus.gov.br/DATASUS/index.php?area=0205) |
| Infant deaths | Deaths under 1 year of age |  |  |
| Neonatal deaths | Deaths in first 28 days of life |  |  |
| Infant mortality rate (IMR) | Infant deaths per 1,000 live births |  |  |
| Neonatal mortality rate (NMR) | Neonatal deaths per 1,000 live births |  |  |
| Low birthweight | Percentage of live births with low birthweight (<2500g) (%) | Ministry of Health DATASUS – SINASC | [http://tabnet.datasus.gov.br/cgi/ deftohtm.exe?sinasc/cnv/nvuf.def](http://tabnet.datasus.gov.br/cgi/deftohtm.exe?sinasc/cnv/nvuf.def) |
| Very low birthweight | Percentage of live births with very low birthweight (<1500g) (%) |  |  |
| Preterm births | Percentage of births preterm (<37 weeks) (%) |  |  |
| Caesarean births | Percentage of births caesarean (%) |  |  |
| Hospital births | Percentage of births in hospital (%) |  |  |
| Mother’s age | Mean mother’s age at birth |  |  |
| Education 0 to 3 years | Percentage of births to mothers with less than 3 years education (%) |  |  |
| Education 4 to 7 years | Percentage of births to mothers with 4 to 7 years education (%) |  |  |
| Education 8 to 11 years | Percentage of births to mothers with 8 to 11 years education (%) |  |  |
| Education 12 or more years | Percentage of mothers with more than 12 years education (%) |  |  |
| No prenatal care | Percentage of births with no prenatal care (%) |  |  |
| 1 to 3 prenatal care visits | Percentage of births with 1-3 prenatal care visits (%) |  |  |
| 4 to 11 prenatal care visits | Percentage of births with 4-6 prenatal care visits (%) |  |  |
| 7 or more prenatal care visits | Percentage of births with 7 or more prenatal care visits (%) |  |  |
| Nurses density | Nurses per 1,000 population | Ministry of Health DATASUS - CNES | [http://tabnet.datasus.gov.br/cgi/ deftohtm.exe?cnes/cnv/prid02br.def](http://tabnet.datasus.gov.br/cgi/deftohtm.exe?cnes/cnv/prid02br.def) |
| Primary care doctor density  Community health worker density  Hospital beds | Primary care doctors per 1,000 population  Community health workers per 100,000 population  Hospital beds per 1,000 population |  |  |
| Infant hospitalisations | Infant hospitalisations per 1,000 births | Ministry of Health DATASUS – SIH | [http://tabnet.datasus.gov.br /cgi/tabcgi.exe?sih/cnv/niuf.def](http://tabnet.datasus.gov.br/cgi/tabcgi.exe?sih/cnv/niuf.def) |
| Private health insurance coverage | Private health insurance plans | Ministry of Health DATASUS - ANS | <http://www.ans.gov.br/anstabnet/> |
| Bolsa Familia stipend | Bolsa Familia stipend per capita (BRL) | MDS | [https://aplicacoes.mds.gov.br /sagi/vis/data3/data-explorer.php](https://aplicacoes.mds.gov.br/sagi/vis/data3/data-explorer.php) |
| Health spending | Municipal health spending per capita (BRL) | SIOPS | [http://www.saude.gov.br/repasses -financeiros/siops/indicadores](http://www.saude.gov.br/repasses-financeiros/siops/indicadores) |

Note: Mortality Information System (Sistema de Informações sobre Mortalidade) (SIM); National Agency for Supplementary Health (Agência Nacional de Saúde) (ANS); National Register of Health Establishments (Cadastro Nacional Estabelecimentos de Saúde) (CNES); Brazilian Institute for Geography and Statistics (Instituto Brasileiro de Geografia e Estastíca) (IBGE); The Ministry of Social Development and Fight Against Hunger (Ministério do Desenvolvimento Social e Combate à Fome) (MDS); Public Health Budget Information System (Sistema Informações sobre Orçamentos Públicos em Saúde) (SIOPS)

* These variables were obtained from the 2000 and 2010 census, and the years 2001-2009 and 2011-2017 were estimated by municipal-level interpolation and extrapolation of trends.

**Appendix S3. Framework illustrating the determinants of infant health in Brazil**


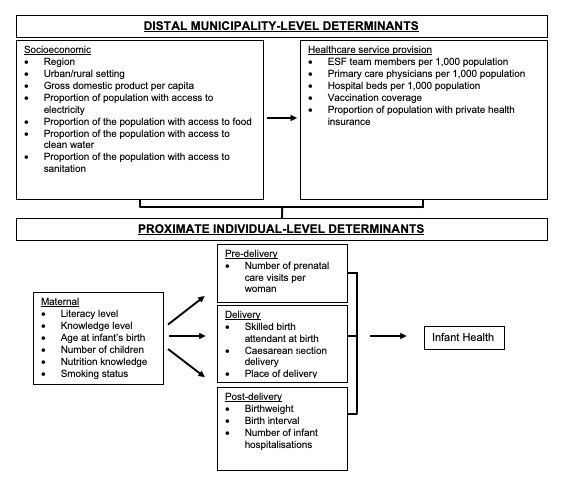


**References**

Khadka KB, Lieberman LS, Giedraitis V, Bhatta L, Pandey G. The socio-economic determinants of infant mortality in Nepal: analysis of Nepal Demographic Health Survey, 2011. BMC Pediatrics. 2015;15(1):1-11.

Macinko J, Guanais FC, Marinho De Souza MDF. Evaluation of the impact of the Family Health Program on infant mortality in Brazil, 1990-2002. Journal of Epidemiology and Community Health. 2006;60(1):13–19.

Mosley WH, Chen LC. An analytical framework for the study of child survival in developing countries. Population and Development Review. 1984;10(1):25-45.

**Appendix S4. Descriptive statistics in PMM receiving and non-PMM receiving municipalities**

|  | **Municipalities receiving PMM  doctors***(N=4,660)* | | **Municipalities not receiving PMM doctors***(N=905)* | |
| --- | --- | --- | --- | --- |
|  | **Mean** | **SD** | **Mean** | **SD** |
| Population | 40999.45 | 228418.10 | 7337.24 | 9412.25 |
| GDP per capita (BRL) | 15844.60 | 17279.77 | 18401.41 | 25192.06 |
| Income per capita (BRL) | 524.07 | 269.66 | 575.19 | 256.51 |
| Gini index | 0.49 | 0.08 | 0.45 | 0.08 |
| Households without electricity (%) | 2.20 | 5.79 | 1.55 | 4.98 |
| Households with inadequate sanitation (%) | 9.46 | 13.80 | 4.47 | 9.45 |
| Urbanisation rate (%) | 64.63 | 22.31 | 64.45 | 21.40 |
| Illiteracy rate (15+ years) (%) | 15.07 | 9.66 | 13.26 | 8.72 |
| Proportion of population with per capita income under 0.25 minimum wage (%) | 20.33 | 18.17 | 13.87 | 15.23 |
| Bolsa Familia stipend per capita (BRL) | 161.55 | 133.89 | 129.51 | 118.60 |
| Health expenditure per capita (BRL) | 468.59 | 262.72 | 637.04 | 349.86 |
| Private health insurance plans per capita | 0.08 | 0.13 | 0.08 | 0.12 |
| Hospital beds per 1,000 population | 1.80 | 2.04 | 1.28 | 2.24 |
| Nurses per 1,000 population | 0.65 | 0.37 | 0.70 | 0.40 |
| Proportion of births low birthweight (%) | 7.66 | 3.13 | 7.86 | 4.59 |
| Proportion of births very low birthweight (%) | 1.03 | 1.08 | 1.06 | 1.71 |
| Infants hospitalised per 1000 live births | 189.43 | 115.95 | 196.89 | 131.40 |
| Neonatal mortality rate (NMR) | 9.82 | 10.27 | 9.51 | 15.42 |
| Infant mortality rate (IMR) | 14.20 | 12.61 | 13.51 | 18.20 |
| Mean municipal mother’s age | 25.41 | 1.44 | 25.70 | 1.69 |
| Proportion of births to mothers with l0 to 3 years education (%) | 7.92 | 7.90 | 5.92 | 6.76 |
| Proportion of births to mothers with 4 to 7 years education (%) | 27.74 | 11.11 | 25.27 | 12.23 |
| Proportion of births to mothers with 8 to 11 years education (%) | 49.64 | 14.53 | 52.60 | 15.27 |
| Proportion of births to mothers with more than 12 years education (%) | 12.34 | 7.70 | 14.02 | 9.07 |
| Proportion of births with no prenatal care (%) | 1.82 | 3.32 | 1.13 | 2.49 |
| Proportion of births with 1-3 prenatal care visits (%) | 6.28 | 6.27 | 4.15 | 4.99 |
| Proportion of births with 4-6 prenatal care visits (%) | 28.00 | 15.05 | 23.08 | 14.52 |
| Proportion of births with 7 or more prenatal care visits (%) | 63.20 | 20.41 | 71.07 | 18.13 |

Sources: Brazilian Ministry of Health DATASUS website; Brazilian Institute for Geography and Statistics (IBGE); Ministry of Social Development and Fight Against Hunger (MDS); Information System for Public Health Budget (SIOPS)

**Appendix S5. Trends in mean rate of IMR in PMM-receiving and non-PMM receiving municipalities**


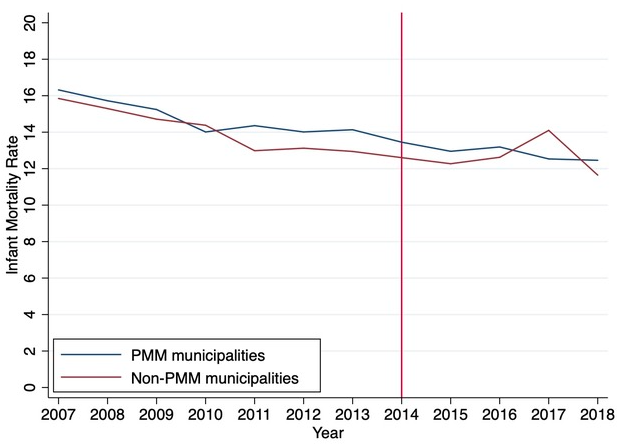


Source: Brazilian Ministry of Health DATASUS website

**Appendix S6. Trends in mean rate of NMR in PMM-receiving and non-PMM receiving municipalities**


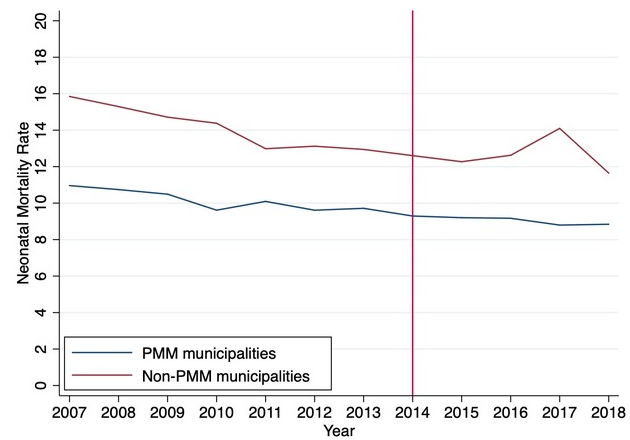


Source: Brazilian Ministry of Health DATASUS website

**Appendix S7: Effect of PMM density on IMR and NMR on aggregate by year since programme introduction**

|  | **IMR** | **95% CI** | **NMR** | **95% CI** |
| --- | --- | --- | --- | --- |
| **Years after PMM introduction** |  |  |  |  |
| 0 | 0.04 | -0.19,0.27 | 0.11 | -0.10,0.32 |
| 1 | -0.01 | -0.17,0.15 | 0.01 | -0.14,0.15 |
| 2 | -0.04 | -0.20,0.12 | -0.04 | -0.17,0.08 |
| 3 | -0.02 | -0.24,0.19 | -0.08 | -0.19,0.03 |
| 4 | 0.01 | -0.13,0.15 | 0.03 | -0.10,0.15 |
| 5 | -0.15 | -0.44,0.14 | -0.05 | -0.29,0.18 |

Notes: * p<0.05, ** p<0.01, *** p<0.001. All models applied inverse probability of treatment weighting with regression adjustment (IPTW-RA). Primary outcome variables: infant mortality rate (IMR) and neonatal mortality rate (NMR). Intermediate outcome variables: infants hospitalised per 1,000 live births; seven or more prenatal care visits; and proportion of infants born with a low birthweight (%). Models adjusted for GDP per capita (BRL), income per capita (BRL), Gini coefficient, proportion of households with inadequate sanitation (%), proportion of households with no electricity (%), proportion of population living in urban areas (%), proportion of population illiterate above the age of 15 (%), proportion of the population with per capita income under 0.25 minimum wage (%), Bolsa Familia stipend (BRL), private health insurance plans per capita, heath expenditure per capita (BRL), hospital beds per 1,000 population, nurses per 1,000 population, mean municipal mother’s age, proportion of mothers with zero to three years of education (%), proportion of mothers with four to seven years of education (%), proportion of mothers with eight to eleven years of education (%), proportion of mothers with more than twelve years of education (%), and municipality and time fixed effects.

**Appendix S8. Effect of PMM density on intermediate outcome variables (1)**

|  | **Low birth weight** | **95% CI** | **Very low birthweight** | **95% CI** | **Infants hospitalised** | **95% CI** |
| --- | --- | --- | --- | --- | --- | --- |
| PMM density per 1,000 population | 0.03* | 0.00,0.05 | 0.00 | -0.01,0.01 | 0.37 | -0.37,1.11 |

Notes: * p<0.05, ** p<0.01, *** p<0.001. All models applied inverse probability of treatment weighting with regression adjustment (IPTW-RA). Intermediate outcome variables: infants hospitalised per 1,000 live births; proportion of infants born with a low birthweight (%); and proportion of infants born with a very low birthweight (%). Models adjusted for GDP per capita (BRL), income per capita (BRL), Gini coefficient, proportion of households with inadequate sanitation (%), proportion of households with no electricity (%), proportion of population living in urban areas (%), proportion of population illiterate above the age of 15 (%), proportion of the population with per capita income under 0.25 minimum wage (%), Bolsa Familia stipend (BRL), private health insurance plans per capita, heath expenditure per capita (BRL), hospital beds per 1,000 population, nurses per 1,000 population, mean municipal mother’s age, proportion of mothers with zero to three years of education (%), proportion of mothers with four to seven years of education (%), proportion of mothers with eight to eleven years of education (%), proportion of mothers with more than twelve years of education (%), and municipality and time fixed effects.

**Appendix S9. Effect of PMM density on intermediate outcome variables on aggregate (2)**

|  | **No prenatal care visits** | **95% CI** | **1-3 prenatal care visits** | **95% CI** | **4-6 prenatal care visits** | **95% CI** | **≤7 prenatal care visits** | **95% CI** |  |
| --- | --- | --- | --- | --- | --- | --- | --- | --- | --- |
| PMM density per 1,000 population | 0.00 | -0.01,0.01 | -0.01 | -0.02,0.01 | -0.04 | -0.10,0.02 | 0.04 | -0.02,0.11 | |

Notes: * p<0.05, ** p<0.01, *** p<0.001. All models applied inverse probability of treatment weighting with regression adjustment (IPTW-RA). Intermediate outcome variables: proportion of births with no prenatal care visits (%); proportion of births with 1-3 prenatal care visits; proportion of births with 4-6 prenatal care visits; proportion of births with seven or more prenatal care visits. Models adjusted for GDP per capita (BRL), income per capita (BRL), Gini coefficient, proportion of households with inadequate sanitation (%), proportion of households with no electricity (%), proportion of population living in urban areas (%), proportion of population illiterate above the age of 15 (%), proportion of the population with per capita income under 0.25 minimum wage (%), Bolsa Familia stipend (BRL), private health insurance plans per capita, heath expenditure per capita (BRL), hospital beds per 1,000 population, nurses per 1,000 population, mean municipal mother’s age, proportion of mothers with zero to three years of education (%), proportion of mothers with four to seven years of education (%), proportion of mothers with eight to eleven years of education (%), proportion of mothers with more than twelve years of education (%), and municipality and time fixed effect.

|  | **IMR** | **95% CI** | **NMR** | **95% CI** | **Infants hospitalised** | **95% CI** | **≤7 prenatal care visits** | **95% CI** | **Low birth weight** | **95% CI** |
| --- | --- | --- | --- | --- | --- | --- | --- | --- | --- | --- |
| **Quintiles of urbanisation** |  |  |  |  |  |  |  |  |  |  |
| Q1  (3.50-43.36) | -0.07 | -0.23,0.10 | -0.08 | -0.22,0.07 | 1.13 | -0.31,2.59 | -0.01 | -0.11,0.08 | 0.05 | -0.00,0.10 |
| Q2  (42.37-59.05) | 0.10 | -0.09,0.29 | 0.07 | -0.09,0.24 | -0.22 | -1.61,1.17 | 0.05 | -0.09,0.19 | 0.03 | -0.02,0.08 |
| Q3 (59.06-73.10) | 0.01 | -0.22,0.24 | -0.02 | -0.18,0.15 | 0.01 | -1.10,1.12 | 0.12 | -0.07,0.29 | -0.02 | -0.06,0.02 |
| Q4 (73.11-86.66) | -0.24 | -0.50,0.02 | -0.07 | -0.28,0.13 | -1.19 | -2.86,0.49 | 0.08 | -0.06,0.23 | 0.01 | -0.05,0.07 |
| Q5 (86.67-100) | 0.38 | -0.13,0.90 | 0.40 | -0.13,0.93 | -1.14 | -3.83,1.54 | -0.04 | -0.23,0.14 | -0.01 | -0.08,0.07 |

**Appendix S10. Effect of PMM density on primary and intermediate outcomes for subgroups of municipal urbanisation rate**

Notes: * p<0.05, ** p<0.01, *** p<0.001. All models applied inverse probability of treatment weighting with regression adjustment (IPTW-RA). Primary outcome variables: infant mortality rate (IMR) and neonatal mortality rate (NMR). Intermediate outcome variables: infants hospitalised per 1,000 live births; seven or more prenatal care visits; and proportion of infants born with a low birthweight (%). Models adjusted for GDP per capita (BRL), income per capita (BRL), Gini coefficient, proportion of households with inadequate sanitation (%), proportion of households with no electricity (%), proportion of population living in urban areas (%), proportion of population illiterate above the age of 15 (%), proportion of the population with per capita income under 0.25 minimum wage (%), Bolsa Familia stipend (BRL), private health insurance plans per capita, heath expenditure per capita (BRL), hospital beds per 1,000 population, nurses per 1,000 population, mean municipal mother’s age, proportion of mothers with zero to three years of education (%), proportion of mothers with four to seven years of education (%), proportion of mothers with eight to eleven years of education (%), proportion of mothers with more than twelve years of education (%), and municipality and time fixed effects.

**Appendix S11. Effect of PMM density on primary and intermediate outcomes for subgroups of municipal poverty rate**

|  | | **IMR** | **95% CI** | **NMR** | **95% CI** | **Infants hospitalised** | **95% CI** | **≤7 prenatal care visits** | **95% CI** | **Low birth weight** | **95% CI** |
| --- | --- | --- | --- | --- | --- | --- | --- | --- | --- | --- | --- |
| **Quintiles of poverty** | |  |  |  |  |  |  |  |  |  |  |
| Q1  (0.18-3.68) | 0.12 | | -0.08,0.31 | 0.16 | -0.01,0.33 | 1.64* | 0.08,3.21 | 0.05 | -0.06,0.16 | -0.08* | -0.01-,0.14 |
| Q2  (3.69-8.21) | -0.14 | | -0.33,0.06 | -0.10 | -0.28,0.08 | -0.34 | -1.46,0.79 | 0.02 | -0.08,0.12 | 0.00 | -0.04,0.04 |
| Q3  (8.22-20.94) | 0.03 | | -0.20,0.26 | -0.01 | -0.16,0.14 | 0.19 | -1.53,1.92 | 0.02 | -0.13,0.17 | 0.04 | -0.01,0.09 |
| Q4 (20.95-36.42) | -0.12 | | -0.30,0.06 | -0.13 | -0.32,0.05 | 0.80 | -0.40,1.99 | -0.01 | -0.19,0.17 | 0.01 | -0.04,0.06 |
| Q5  (36.43-78.79) | 0.11 | | -0.11,0.33 | 0.03 | -0.15,0.22 | -1.46 | -4.40,1.47 | 0.17 | -0.06,0.40 | -0.01 | -0.06,0.03 |

Notes: * p<0.05, ** p<0.01, *** p<0.001. All models applied inverse probability of treatment weighting with regression adjustment (IPTW-RA). Primary outcome variables: infant mortality rate (IMR) and neonatal mortality rate (NMR). Intermediate outcome variables: infants hospitalised per 1,000 live births; seven or more prenatal care visits; and proportion of infants born with a low birthweight (%). Models adjusted for GDP per capita (BRL), income per capita (BRL), Gini coefficient, proportion of households with inadequate sanitation (%), proportion of households with no electricity (%), proportion of population living in urban areas (%), proportion of population illiterate above the age of 15 (%), proportion of the population with per capita income under 0.25 minimum wage (%), Bolsa Familia stipend (BRL), private health insurance plans per capita, heath expenditure per capita (BRL), hospital beds per 1,000 population, nurses per 1,000 population, mean municipal mother’s age, proportion of mothers with zero to three years of education (%), proportion of mothers with four to seven years of education (%), proportion of mothers with eight to eleven years of education (%), proportion of mothers with more than twelve years of education (%), and municipality and time fixed effects.

**Appendix S12: Effect of PMM density on IMR and NMR for municipalities with highest IMR at baseline (25.24-209.3 infant deaths per 1,000 live births)**

|  | **IMR** | **95% CI** | **NMR** | **95% CI** |
| --- | --- | --- | --- | --- |
| **Years after PMM introduction** |  |  |  |  |
| 0 | -0.61** | -0.99,0.-24 | -0.30 | -0.64,0.04 |
| 1 | -0.31* | -0.58,0.05 | -0.28** | -0.47,-0.08 |
| 2 | -0.23 | -0.51,0.05 | -0.15 | -0.39,0.09 |
| 3 | -0.09 | -0.40,0.22 | -0.09 | -0.35,0.16 |
| 4 | 0.00 | -0.29,0.29 | 0.10 | -0.17,0.37 |
| 5 | -0.39 | -0.87,0.10 | -0.30 | -0.58,-0.01 |

Notes: * p<0.05, ** p<0.01, *** p<0.001. All models applied inverse probability of treatment weighting with regression adjustment (IPTW-RA). Primary outcome variables: infant mortality rate (IMR) and neonatal mortality rate (NMR). Intermediate outcome variables: infants hospitalised per 1,000 live births; seven or more prenatal care visits; and proportion of infants born with a low birthweight (%). Models adjusted for GDP per capita (BRL), income per capita (BRL), Gini coefficient, proportion of households with inadequate sanitation (%), proportion of households with no electricity (%), proportion of population living in urban areas (%), proportion of population illiterate above the age of 15 (%), proportion of the population with per capita income under 0.25 minimum wage (%), Bolsa Familia stipend (BRL), private health insurance plans per capita, heath expenditure per capita (BRL), hospital beds per 1,000 population, nurses per 1,000 population, mean municipal mother’s age, proportion of mothers with zero to three years of education (%), proportion of mothers with four to seven years of education (%), proportion of mothers with eight to eleven years of education (%), proportion of mothers with more than twelve years of education (%), and municipality and time fixed effects.

**Appendix S13. Effect of PMM density on primary and intermediate outcomes for subgroups of municipal non-PMM doctor density**

|  | **IMR** | **95% CI** | **NMR** | **95% CI** | **Infants hospitalised** | **95% CI** | **≤7 prenatal care visits** | **95% CI** | **Low birth weight** | **95% CI** |  |
| --- | --- | --- | --- | --- | --- | --- | --- | --- | --- | --- | --- |
| **Quintiles of non PMM primary care doctor density (2012)** |  |  |  |  |  |  |  |  |  |  |  |
| Q1  (0-1.08) | -0.13 | -1.10,0.84 | 0.10 | -0.68,0.88 | -1.78 | -11.17,7.61 | 0.61 | -1.09,2.32 | -0.11 | -0.33,0.10 | |
| Q2  (1.09-2.66) | 0.77** | 0.21,1.34 | 0.59** | 0.17,1.01 | -0.30 | -6.32,5.71 | 0.52 | -0.41,1.44 | 0.08 | -0.06,0.23 | |
| Q3  (2.67-5.43) | 0.34* | 0.00,0.68 | 0.34* | 0.04,0.65 | 1.33 | -1.89,4.54 | 0.32 | -0.14,0.78 | 0.07 | -0.01,0.14 | |
| Q4  (5.44-11.63) | -0.03 | -0.26,0.19 | -0.02 | -0.19,0.15 | -1.30 | -3.56,0.96 | 0.13 | -0.07,0.34 | -0.00 | -0.06,0.06 | |
| Q5  (11.64-157.39) | -0.04 | -0.16,0.09 | -0.03 | -0.13,0.07 | -0.16 | -0.71,1.04 | 0.08* | 0.01,0.16 | 0.03 | -0.00,0.06 | |

Notes: * p<0.05, ** p<0.01, *** p<0.001. All models applied inverse probability of treatment weighting with regression adjustment (IPTW-RA). Primary outcome variables: infant mortality rate (IMR) and neonatal mortality rate (NMR). Intermediate outcome variables: infants hospitalised per 1,000 live births; seven or more prenatal care visits; and proportion of infants born with a low birthweight (%). Models adjusted for GDP per capita (BRL), income per capita (BRL), Gini coefficient, proportion of households with inadequate sanitation (%), proportion of households with no electricity (%), proportion of population living in urban areas (%), proportion of population illiterate above the age of 15 (%), proportion of the population with per capita income under 0.25 minimum wage (%), Bolsa Familia spend (BRL), private health insurance plans per capita, heath expenditure per capita (BRL), hospital beds per 1,000 population, nurses per 1,000 population, mean municipal mother’s age, proportion of mothers with zero to three years of education (%), proportion of mothers with four to seven years of education (%), proportion of mothers with eight to eleven years of education (%), proportion of mothers with more than twelve years of education (%), and municipality and time fixed effects.

**Appendix S14. Effect of PMM density on primary and intermediate outcomes for subgroups of municipal nurse density**

|  | **IMR** | **95% CI** | **NMR** | **95% CI** | **Infants hospitalised** | **95% CI** | **≤7 prenatal care visits** | **95% CI** | **Low birth weight** | **95% CI** |
| --- | --- | --- | --- | --- | --- | --- | --- | --- | --- | --- |
| **Quintiles of nurse density 2012** |  |  |  |  |  |  |  |  |  |  |
| Q1  (0-0.38) | -0.01 | -0.24,0.22 | 0.05 | -0.15,0.25 | -0.40 | -2.43,1.63 | 0.08 | -0.12,0.27 | 0.01 | -0.04,0.07 |
| Q2 (0.39-0.50) | 0.01 | -0.25,0.27 | -0.06 | -0.25,0.14 | -0.19 | -1.76,1.39 | -0.05 | -0.21,0.11 | 0.05 | -0.01,0.12 |
| Q3 (0.51-0.63) | -0.18 | -0.49,0.13 | -0.18 | -0.38,0.02 | 1.26 | -1.14,3.67 | 0.05 | -0.10,0.20 | 0.03 | -0.06,0.11 |
| Q4 (0.64-0.82) | 0.01 | -0.16,0.17 | 0.01 | -0.09,0.11 | 0.52 | -0.58,1.62 | 0.06 | -0.09,0.21 | 0.03 | -0.00,0.07 |
| Q5  (0.83-4.15) | 0.03 | -0.14,0.20 | 0.08 | -0.08,0.23 | 0.25 | -0.96,1.46 | 0.06 | -0.05,0.17 | 0.02 | -0.02,0.07 |

Notes: * p<0.05, ** p<0.01, *** p<0.001. All models applied inverse probability of treatment weighting with regression adjustment (IPTW-RA). Primary outcome variables: infant mortality rate (IMR) and neonatal mortality rate (NMR). Intermediate outcome variables: infants hospitalised per 1,000 live births; seven or more prenatal care visits; and proportion of infants born with a low birthweight (%). Models adjusted for GDP per capita (BRL), income per capita (BRL), Gini coefficient, proportion of households with inadequate sanitation (%), proportion of households with no electricity (%), proportion of population living in urban areas (%), proportion of population illiterate above the age of 15 (%), proportion of the population with per capita income under 0.25 minimum wage (%), Bolsa Familia stipend (BRL), private health insurance plans per capita, heath expenditure per capita (BRL), hospital beds per 1,000 population, nurses per 1,000 population, mean municipal mother’s age, proportion of mothers with zero to three years of education (%), proportion of mothers with four to seven years of education (%), proportion of mothers with eight to eleven years of education (%), proportion of mothers with more than twelve years of education (%), and municipality and time fixed effects.

**Appendix S15. Effect of PMM density on primary and intermediate outcomes for subgroups of municipal CHW density**

|  | **IMR** | **95% CI** | **NMR** | **95% CI** | **Infants hospitalised** | **95% CI** | **≤7 prenatal care visits** | **95% CI** | **Low birth weight** | **95% CI** |
| --- | --- | --- | --- | --- | --- | --- | --- | --- | --- | --- |
| **Quintiles of CHW density** |  |  |  |  |  |  |  |  |  |  |
| Q1  (0-1.75) | -0.12 | -0.73,0.49 | -0.15 | -0.77,0.47 | 0.18 | -2.90,3.27 | -0.09 | -0.35,0.17 | 0.02 | -0.04,0.08 |
| Q2  (1.76-2.26) | -0.20 | -0.41,0.02 | -0.01 | -0.24,0.21 | -1.47 | -3.92,0.97 | 0.00 | -0.22,0.23 | 0.02 | -0.04,0.08 |
| Q3  (2.27-2.51) | -0.11 | -0.27,0.05 | -0.04 | -0.18,0.10 | 0.90 | -0.61,2.40 | -0.04 | -0.16,0.08 | 0.02 | -0.04,0.07 |
| Q4 (2.52-2.83) | 0.08 | -0.16,0.32 | 0.01 | -0.19,0.22 | 0.38 | -1.52,2.27 | 0.03 | -0.10,0.16 | 0.02 | -0.04,0.08 |
| Q5  (2.84-7.26) | 0.05 | -0.11,0.21 | 0.02 | -0.09,0.13 | 0.27 | -0.84,1.38 | 0.13* | 0.01,0.24 | 0.04 | -0.01,0.08 |

Notes: * p<0.05, ** p<0.01, *** p<0.001. All models applied inverse probability of treatment weighting with regression adjustment (IPTW-RA). Primary outcome variables: infant mortality rate (IMR) and neonatal mortality rate (NMR). Intermediate outcome variables: infants hospitalised per 1,000 live births; seven or more prenatal care visits; and proportion of infants born with a low birthweight (%). Models adjusted for GDP per capita (BRL), income per capita (BRL), Gini coefficient, proportion of households with inadequate sanitation (%), proportion of households with no electricity (%), proportion of population living in urban areas (%), proportion of population illiterate above the age of 15 (%), proportion of the population with per capita income under 0.25 minimum wage (%), Bolsa Familia stipend (BRL), private health insurance plans per capita, heath expenditure per capita (BRL), hospital beds per 1,000 population, nurses per 1,000 population, mean municipal mother’s age, proportion of mothers with zero to three years of education (%), proportion of mothers with four to seven years of education (%), proportion of mothers with eight to eleven years of education (%), proportion of mothers with more than twelve years of education (%), and municipality and time fixed effects.
